# Children as Alibi Corroborators for Adults

**DOI:** 10.1111/nyas.70179

**Published:** 2026-01-02

**Authors:** Heather L. Price, Angela D. Evans, Emily A. Nevokshonoff, Andre Kehn, Jennica Wlodarczyk

**Affiliations:** ^1^ Department of Psychology Thompson Rivers University Kamloops British Columbia Canada; ^2^ Department of Psychology Brock University St. Catharines Ontario Canada; ^3^ Department of Psychology University of British Columbia Vancouver British Columbia Canada; ^4^ Department of Psychology University of North Dakota Grand Forks North Dakota USA

**Keywords:** alibi, alibi corroborator, child, memory, memory for people, witness

## Abstract

Despite how frequently adults are alone with children, we know little about children's ability to corroborate alibis. In two studies, we investigated children's ability to act as alibi corroborators. In both studies, two visitors (one male, one female) attended children's summer camps to present science activities to the children. In the pilot study (*N* = 83; *M*_age_ = 7.1 years), for half of the children, the female researcher left the room for one of the activities. Children were then interviewed about the adults’ whereabouts either immediately or 1 day later. In the main study (*N* = 147; *M*_age_ = 9.40), the female researcher left the room for one activity in a more salient manner, and all children were interviewed 3 days later. Across both studies, though there was substantial variability, many children did not report that the female researcher left (pilot study, 82%; main study, 32%), despite direct questions about her presence. All inaccurate reports of an adult leaving were in response to the most direct (yes/no) question. These findings suggest that children are largely accurate in corroborating an alibi for someone who did not leave, but many children err when someone does leave. The present studies have implications for how to question children about an adult's whereabouts.

## Graphical abstract

We investigated children's ability to report that an adult had left the room and was thus not present for a portion of a group activity. Most children were able to report when an adult stayed in the room, but errors were much more common in noticing and reporting that an adult had left the room. These findings have implications for children as alibi corroborators of adults’ activities.

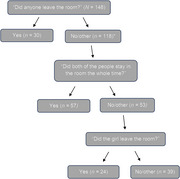

## 1 Introduction

When a person is accused of committing a crime, a central defense available to them is an alibi. An alibi is a statement of the accused person's whereabouts that provides evidence that they could not have committed the crime because they were elsewhere. In most cases, alibis are corroborated by person evidence (i.e., an alibi corroborator or witness) rather than physical evidence (e.g., video surveillance, receipts [1, 2, 3]). Most people who have been wrongfully convicted of crimes and subsequently exonerated have been shown to have had alibis for the time of the crime, but their alibis were not believed [4, 5, 6]. As a result, the extant alibi literature has largely focused on the credibility of alibi witnesses in an attempt to understand why an alibi might not be believed [7]. Through this research, we have come to understand that people who have a close relationship with an accused person and those who are otherwise perceived as potentially motivated to lie [8] are less likely to be believed [7]. A further important question that has been a more substantive focus of recent empirical attention is how well a potential alibi witness remembers the actions of the accused person. That is, even if an alibi corroborator is believable, how likely are they to be accurate in their recollection? And, what factors might influence the accuracy of an alibi corroborator? This question is important in terms of the ability to either corroborate or contradict an alibi, as both speak to the reliability of a corroborator. In the current research, we consider the accuracy of a common potential alibi corroborator—a child [9].

As many parents and adults who regularly interact with children will know, adults are often alone with children. If an adult is then accused of a crime that took place during that time, the child may be their only potential alibi corroborator. Some research has indicated that children are perceived as more credible than adults who serve as alibi corroborators. In one study, Dahl and Price [10, 11] had children (6 years old) and adults (25 years old) each provide an alibi witness statement that spanned an entire day for a person accused of robbery. They found that children were perceived as more credible and believable than adult alibi corroborators. Relying on past predictors of children's credibility [12, 13], the authors speculated that when honesty is more salient to children's evidence, particularly young children, they are more likely to be believed than adults. However, when accuracy is more salient to the alibi (e.g., when time precision is required), adults may be more credible than children. Consistent with this possibility, children may be more credible than adults in circumstances in which accuracy is not as salient (as is the case when time precision is not required, like in a full‐day alibi [14]). The relative credibility of child alibi corroborators is complex, however, and has previously produced inconsistent findings. For example, Eastwood and colleagues [15] found that adults were perceived as more credible than children as alibi corroborators by 90% of their participants who evaluated alibis requiring time precision, with many participant comments reflecting concerns about children's susceptibility to external influences on reporting. Yet, other studies have found no differences between children and adults [14, 16, 17]. The most common finding, however, reflects the findings of the earliest work: when honesty is salient, young children are seen as credible, and when accuracy is salient, their credibility suffers.

It follows from this research that it is important to establish what children will actually be able to remember about an adult's whereabouts. However, there is no research that directly addresses the question of children's ability to act as an alibi corroborator by remembering the presence, timing, and actions of an adult. In the current research, we isolated the ability to remember the actions of others while minimizing the motivation to potentially lie for others. As such, we focus on the circumstance in which a child is asked about the actions of a person the child met very recently (avoiding the potential motivation to lie for someone they are close to [8]).

Although researchers have yet to examine children's alibi accuracy, research has addressed the question of an adult's ability. For example, some research has examined adults’ ability to generate their *own* alibi, remembering prior whereabouts during specific time periods, and has generally found that this is difficult to do. For example, Olson and Charman [18] found that over one‐third of people asked to generate an alibi were mistaken in the details they provided, with more mistakes observed with longer durations from the day of interest to recall [19]. It is not surprising that recalling such details is difficult, and volumes of memory research confirm expected challenges in this task—perhaps particularly so when applied to recalling details of one's own life that is often filled with days with similar structure (see Crozier and colleagues [20] for a discussion of basic memory processes and alibis).

Despite the difficulties in recalling one's own behavior that are evidenced in this prior research, there are factors that might make generating a person's *own* alibi easier (e.g., motivation, meaningfulness of actions, access to the context of the actions or behavior) than confirming the alibi of *another person* (e.g., reduced self‐relevance [21]). To be able to later recall details of a person's activities, a potential alibi corroborator must attend to and encode the person's activities at a particular time and place, they must remember this information over time, and they must successfully retrieve this information at a later time when a questioner requests it. Success at each of these stages may be less likely when a potential alibi corroborator does not know that this information will become important at a later time [22].

Based on these prior findings, if a child is unable to corroborate an adult's true alibi, it is likely to be the result of at least one of three possibilities: encoding failure, retrieval failure, or social influences. First, a child may simply not have encoded the information needed to corroborate the alibi. The child may not have noticed the adult's activities, the presence or absence of the adult, or they may not have attended to the time and place of the activities. And, in keeping with the prior literature on children's recall of temporal information, some children may not have the level of understanding of temporal constructs to be able to accurately encode such detail. There is evidence that the cognitive skills involved in recalling past time and place develop throughout childhood [23, 24, 25, 26], and the extant research indicates that providing such temporal detail about personal experiences is very difficult for children to do with specificity and accuracy [27]. Yet, specificity and accuracy are precisely the nature of details required for alibi corroboration. Second, the child may no longer remember or may fail to retrieve the memory of the adult's actions. That is, even if the adult's presence or absence was encoded at the time, the child's memory for the day may have faded or become inaccessible due to forgetting processes (i.e., natural memory decay over time [28]). Similarly, the relevant information may not come to the child's mind because the questioner did not ask a question that adequately informed the child about what information was being requested, thus resulting in retrieval failure. Third, the child may have both encoded and retrieved the memory of the activities, but the child did not report the information to a questioner. This latter option could be because the child felt some social pressure (real or perceived) to withhold the information [8] or that the child did not understand the importance of providing the information. Given all of these factors that may limit a child's ability to provide an alibi, it is likely that children struggle to provide accurate alibi information.

### 1.1 The Present Research

While previous researchers have examined how adults evaluate child alibi corroborators, we know little about how well children are able to act as alibi corroborators to an adult's activities. In two studies (pilot study, main study), we assessed children's ability to serve as an alibi corroborator. Given that no prior research has been conducted on the accuracy of children's ability to provide an alibi, we began by conducting a pilot study to develop a relevant alibi paradigm and assess potential delay manipulations. In the pilot study (*n* = 83), children participated in a series of science activities with two visitors to their science camp. For some children, one of the visitors left the room during one of the activities. Children were then either interviewed directly after the activities or on the following day about the adults’ activities. Based on the pilot study, in the main study (*n* = 143), all children experienced one of the visitors leaving the room, and all interviews took place 3 days following the event. We anticipated that children would struggle to provide information that was specific enough to serve as corroborating alibi evidence, but this research was largely exploratory.

## 2 Pilot Study

### 2.1 Pilot Study Method

#### 2.1.1 Participants and Design

Eighty‐three participants between the ages of 6 and 8 years (M = 7.10, SD = 0.53; 57.8% males, 42.2% females) were recruited through a summer science camp. Parental consent and child assent were provided prior to commencing testing. Children participated in group activity sessions led by two research assistants (one male, one female). The presence of the researchers was manipulated such that for some children, the female research assistant left for 10 min of the 45‐min session (Leave condition: *n* = 43), and for some children, both research assistants stayed for the full session (Stay condition: *n* = 40). The Stay condition (as well as the inclusion of the male who remained for the entire session) allowed for the assessment of whether children could accurately report an alibi for the presence of an adult, as well as whether they would false alarm in response to any of the questions about the research assistants leaving. Either 10–30 min later (no delay: *n* = 39) or 1 day later (1‐day delay; *n* = 44), an interview was conducted with the children about their memory for the research assistants’ whereabouts. Thus, this was a 2 (researcher presence: Leave, Stay) × 2 (delay: no delay, 1‐day delay) between‐subjects design. This project was approved by the University Ethics Board.

#### 2.1.2 Materials and Procedure

Two research assistants (always the same male and female) led a series of four climate‐related activities for approximately 45 min. The research assistants stood in front of the group, and the male research assistant verbally described each activity, while the female research assistant demonstrated the activities. Following the demonstration of each activity, the research assistants distributed materials to the children so they could complete the activity themselves. The research assistants walked around the classroom and provided support to individual children during practice. In the Leave condition, the female research assistant left the room without explanation for 10 min following completion of the first activity. She returned for the beginning of the third activity. In the Stay condition, both research assistants stayed for the full activity session.

Either 10–30 min after the session (no delay condition) or 1 day later (1‐day delay condition), children with parental consent were invited to take part in an individual interview about the event (see Appendix in the Supporting Information for the question script). To begin, children were asked a series of questions about time and sequence of the activity session as part of a separate investigation (e.g., “How long did it take to play all of the climate games?” and “How long ago did you play the climate games?”). Children were then asked the questions of interest for the current study, designed to assess the children's account of who was present during the activities.

Children were posed a series of prompts, beginning with a cued invitation about who was in the room during the climate games (“Tell me which adults were in the room during the climate games.”). Children were then asked, “Did any adults leave the room during the climate games?” If children said yes, they were asked to describe or name the adult(s) who left the room. Questions became increasingly specific about the adult presence in the room and were only asked if the children did not provide a definitive leave/stay response to earlier questions; “There were two people who taught you the climate games. Did both of the people stay in the room the whole time you did the climate games?” Next, “Did the boy who taught you the climate games leave the room?” and “Did the girl who taught you the climate games leave the room?”

If the child indicated that either the male or female research assistant left the room during the activities, they were asked a series of questions to assess their ability to provide alibi corroboration, “How long was s/he gone for? What was happening in the room when s/he left? Where do you think s/he went?” If participants were unable to say how long the person was gone for, they were asked to provide an estimate. Interviews took an average of 10 min.

##### 2.1.2.1 Coding

Children's responses were double‐coded by two research assistants. The actual duration of the research assistant's absence was 10 min, so we considered a range of 5–15 min accurate. Children's responses to the question of what activity took place when the research assistant left were coded according to the activity session script (0 = inaccurate [e.g., described another activity, said I don't know]; 1 = accurately described activity performed with absent; 2 = were vague but correct with children mentioning that it was during an activity, but was not descriptive in the nature of the activity). Children's responses to the question of where the research assistant went during her absence were coded for response themes. Intercoder agreement was very high, with 100% agreement on children's duration estimates, 98.80% agreement on children's reports of the activity taking place when the research assistant left, and 100% agreement on where the research assistant went. The only disagreement was resolved through discussion.

### 2.2 Pilot Study Results

#### 2.2.1 Reports of Leaving

Children's responses about the experimenter's departure from the room were categorized as accurate or inaccurate. Accurate responses to the question of whether or not the female experimenter left the room were either reporting that she left when she actually did or reporting that she did not leave the room when she did not. Any response that indicated that the male left the room was always inaccurate because he always stayed in the room for the full session. In response to the first question of which adults were in the room, many children provided a narrative response that made accuracy assessment challenging (e.g., “some boys and girls”). Thus, we do not analyze responses to this question. Given the low values in some cells and our lack of hypothesized interactions, we focus on main effects.

When responses to all questions were combined, only 13 of the 83 children (15.7%) reported that the female left at some point: 10 were in the Leave condition (23.3% of all 43 Leave condition responses) and three were false alarms in the Stay condition (8% of all 40 Stay condition responses). Recall that children were only asked the increasingly specific questions about the female leaving if they had not previously reported that she left. Children's reports of leaving came in response to all three question types: Did anyone leave the room? (report of female leaving, *n* = 2); Did both people stay in the room the whole time? (report of female leaving *n* = 1); Did the girl who taught you leave the room? (report of female leaving *n* = 10). Notably, all of the children who incorrectly reported that the female left did so in response to the final, most specific question. For the male research assistant, only three children inaccurately reported that he left. As with children's responses to inquiries about the female leaving, all incorrect reports of the male leaving came in response to the final, most specific question. Next, we outline children's response patterns to each of the direct questions about whether the adults stayed or left.

*Did any adult leave the room* *?* All children were asked if any adult left the room, and if they indicated “yes” (*n* = 15), they were asked who. In response to this follow‐up prompt, two children reported that the female research assistant left the room (most others replied with “I don't know” or “I forget”).

*Did both adults stay the whole time?* All children were asked if both the male and female research assistants remained in the room during the complete climate change games (45 min in duration). Seventy children responded with a conclusive “yes” to this question (Leave *n* = 34, 79%; Stay *n* = 36, 90%), one was “not sure” and one said “I think so” (both in the Leave condition), and nine others responded in an uninterpretable way (e.g., off‐topic). Only two children responded by saying “no” (one in each of the Leave/Stay conditions).

*Did the boy leave?* Table 1 shows the distribution of responses across conditions. In response to the question of whether or not the male research assistant left the room during the climate change games, 66 of 83 (79.5%) children accurately reported that he did not leave. Three children inaccurately reported that he left (all in the Stay condition), and 14 children responded with a variation of “I don't know” or provided an uninterpretable response (told a story).

**TABLE 1:** Proportion (number) of children providing each response to the direct question in the pilot study.

		Did female leave?			Did male leave?		
Female presence	Delay	Yes	No	Other	Yes	No	Other
Leave	None	0.21 (4)	0.53 (10)	0.26 (5)	0	0.70 (14)	0.30 (6)
	1‐Day	0.17 (4)	0.71 (17)	0.13 (3)	0	0.83 (19)	0.17 (4)
**Total**		**0.19 (8**)	**0.63 (27)**	**0.19 (8)**	**0**	**0.83 (33)**	**0.23 (10)**
Stay	None	0	0.85 (17)	0.15 (3)	0.05 (1)	0.85 (17)	0.10 (2)
	1‐Day	0.15 (3)	0.75 (15)	0.10 (2)	0.10 (2)	0.80 (16)	0.10 (2)
**Total**		**0.08 (3)**	**0.80 (32)**	**0.13 (5)**	**0.08 (3)**	**0.83 (33)**	**0.10 (4)**

*Did the girl leave?* As can be seen in Table 1, children were much more likely to respond with “no” than any other response type (71% of total responses) in response to the direct query about the female research assistant leaving. Further, a few children in the Leave condition correctly reported that the female researcher left the room in both the No‐Delay (21%) or 1‐Day Delay (17%) condition. This low level of reported “leave” observations led to unequal cell sizes, which made analyses challenging. Thus, we conducted only simple *z*‐tests comparing the proportion of children's responses that were accurate and inaccurate between Leave/Stay conditions. Note that accurate and inaccurate responses do not sum to 100% due to children's “don't know” and other response types. Children were more likely to provide an inaccurate response in the Leave condition (63%) than in the Stay condition (8%), *z* = 6.45, *p* < 0.001, and children provided more accurate responses in the Stay (80%) than Leave condition (19%), *z* = 6.99, *p* < 0.001. Further, children's accurate responses did not differ between the no delay (54%) and 1‐day delay conditions (57%), *z* = 0.32, *p* = 0.75. However, children provided significantly fewer inaccurate responses when there was no delay (26%) versus in the 1‐day delay condition (61%), *z* = 3.13, *p* < 0.01.

#### 2.2.2 Details About Leaving

Given that only 13 children reported that the female research assistant left the room (10 accurately), we were not able to conduct analyses to compare conditions. Thus, we summarize the responses from the children who accurately indicated that the female research assistant left the room and the children who inaccurately reported that either the male or female research assistant left the room.

##### 2.2.2.1 Children Who Accurately Reported Leaving (*n* = 10)

Children's estimates of how long she left the room for (actually 10 min) ranged from 50 s to “5 min or more.” Three children's responses were coded as accurate, and seven children's responses were coded as inaccurate. Most children (*n* = 7) were inaccurate when asked during which activity the research assistant left, but one child was correct, and two children were correct but vague in their descriptions. When asked where they think she went, most children (*n* = 7) speculated by guessing a range of activities that were typical for adults in the camp. That is, they appeared to fill‐in‐the‐blanks of their knowledge by hypothesizing that she went to get more supplies, to speak with another camp employee, or to go to the bathroom. One child provided a response that was difficult to classify, and the remaining two children declined to speculate.

##### 2.2.2.2 Children Who Inaccurately Reported Leaving (*n =* 6)

Three children inaccurately reported that the female left the room, and three children inaccurately reported that the male left the room. Two of these children inaccurately reported that both the male and female left the room. Although there are no “correct” answers to these questions because the research assistants did not actually leave the room in these conditions, we provide these details here to allow for comparison to a child reporting, by chance, the accurate details of the research assistant leaving. All six children were incorrect in their estimates of how long the research assistant was gone for. Most children (*n* = 5) were inaccurate when asked during which activity the research assistant left, but one child reported the activity that took place during the female research assistant's absence, by chance. When asked where the research assistant went, three children reported a reasonable camp activity, two said they didn't know, and one child provided a response that was difficult to classify.

### 2.3 Pilot Study Discussion

Most children were accurate in corroborating an adult's presence in the room; however, despite 43 children experiencing the departure of the female research assistant for almost a quarter of the duration of the experimenters’ visit, only 16% of children reported her departure to an interviewer. We were surprised at how few children reported that the female research assistant left when she did, indeed, leave the room. The role of the female research assistant was high‐profile, instructional, and engaged in the group activity, and her absence meant the male instructor guided the full activity without assistance. Children were prompted with multiple opportunities, including some very direct prompts, to provide this information, but few did.

Although the pilot data did not allow us to disentangle the three possibilities discussed in the introduction for why a child may or may not be able to provide an accurate alibi (encoding, retrieval, social pressures), it is important to consider each, as there are different strategies that can be employed to aid children's reporting depending on whether the inaccuracies result from encoding or retrieval failures or from social pressures. Given the short delay between the event and retrieval, even in the longer delay condition, we think it is unlikely that children's failure to report the research assistant's absence was a retrieval issue (though there was some evidence that the no‐delay condition produced even fewer inaccurate responses than the 1‐day delay condition). Further, there is little reason to believe that social pressures were a concern in the pilot study because children had only just met the research assistant, there were no consequences tied to the ability to report her absence, and the context of the camp made it normal for an adult to leave the room to get supplies or go to the bathroom. Thus, in the main study, we addressed the possibility of impoverished encoding by increasing the salience of the female's absence. In the main study, the female left the room for several minutes and returned with a large and notable item used centrally in the subsequent demonstration (a 100‐pound geode which was wheeled in on a trolley cart).

## 3 Main Study

The main study employed the same basic methodology as the pilot study, with a few modifications. Our most substantive change was the increase in the salience of the female research assistant's absence to address children's potential failure to encode the event. The event was shorter in duration (approximately 10 min, compared to 45 min in the pilot study, due to camp requirements), and the female research assistant left for a shorter period of time (3−4 min; though a similar proportion of the overall event). We did not include a Leave/Stay manipulation and instead focused on the “Leave” condition because a few children in the pilot study inaccurately reported that an adult left when they did not, and the rates of false alarms were the same for the female in the Stay condition and for the male, who never left. We thus excluded the Leave/Stay manipulation and retained the male who stayed for the full session to assess accuracy for when someone remained in the room, as well as false alarms within subjects. We also did not include a manipulation of delay and instead implemented a 3‐day delay to recall to increase the likelihood that some children may forget the now more salient absence of the female research assistant. Finally, given camp availability, the age range for participants in the main study was much wider than the pilot study (pilot study 6‐to‐8 years; main study 6‐to‐13 years), which allowed us to explore age differences. One hundred and forty‐eight children (*M*_age_ = 9.40 years, SD = 1.74; six 6‐year‐olds; 19 7‐year‐olds; 20 8‐year‐olds; 29 9‐year‐olds; 33 10‐year‐olds; 20 11‐year‐olds; 15 12‐year‐olds; four 13‐year‐olds) were recruited from a summer science camp. All children had parental consent and verbally assented to participate.

### 3.1 Coding

Children's responses were coded using the same coding scheme as in the pilot study. However, to determine duration accuracy, the range of 2–5 min was used due to the shorter duration of the female's absence. Further, the responses to the question of where the research assistant went were coded into themes (as in the pilot study) as well as accuracy (correct response to retrieve the geode) since the whereabouts of the female was now clear to the children. Intercoder agreement was again very high, with 100% agreement on duration, 96.6% agreement on the activity taking place when the research assistant left, and 99.32% agreement on where the research assistant went. All disagreements were resolved through discussion.

### 3.2 Main Study Results

**FIGURE 1:**
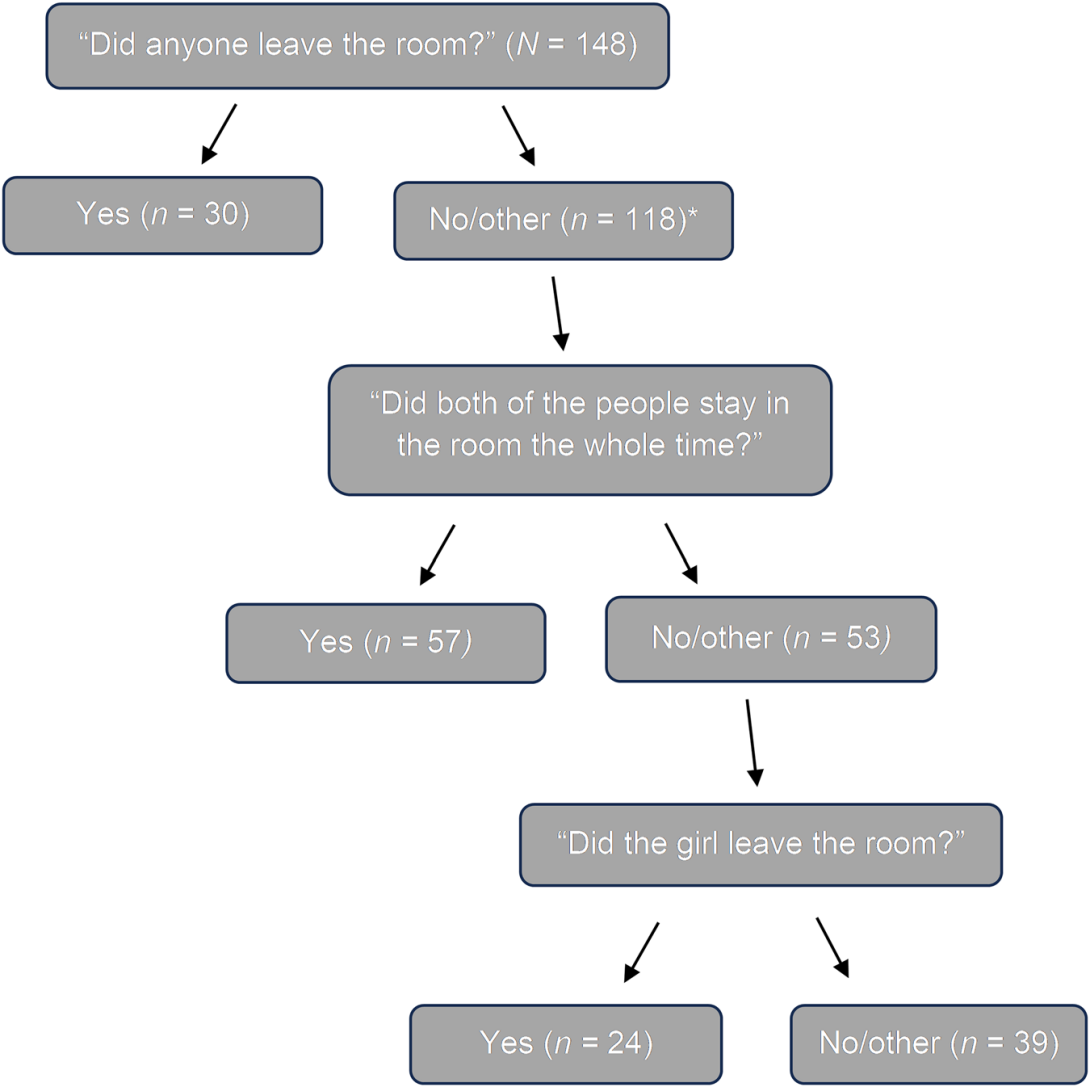
Children's accurate reports of the female research assistant's absence in the main study. * Note that although 118 children responded “no” to the first question, eight of these children were not asked the next question due to interviewer error.

When responses to all questions were combined, 101 of the 148 children (68.2%) reported that the female left. Children's reports of leaving came in response to all three question types: Did anyone leave the room? (report of female leaving in response to follow‐up “who” question, *n* = 30); Did both people stay in the room the whole time? (report of female leaving in response to follow‐up “who” question *n* = 47); Did the girl who taught you leave the room? (report of female leaving *n* = 24). See Figure 1 for a visual depiction of children's reports of the female research assistant's absence in response to each question. For the male research assistant, seven (4.7%) children falsely reported that he left.

To explore potential age differences in children's accuracy in reporting the female research assistant's absence, we conducted a binary logistic regression analysis on children's accurate responses (0 = inaccurate, 1 = accurate), with accurate children reporting at least once that the female research assistant left the room, and age in years as the predictor variable. There was no significant effect of age, *χ* ^2^ (1) = 0.30, *p* = 0.586, Nagelkerke *R* ^2^ = 0.003. Thus, in the remaining results, we collapse across age.

#### 3.2.1 Reports of Leaving

*Did any adult leave the room?* All children were asked if any adult left the room, and if they indicated “yes,” they were asked who. In response to this follow‐up prompt, 30 (20.3%) children reported that the female research assistant left the room.

*Did both adults stay the whole time?* One hundred and ten children were asked if both the male and female research assistants remained in the room during the full duration of the science games (10 min). Fifty‐seven (50.9%) children responded with a conclusive “yes” to this question, and 53 (47.3%) children responded with a conclusive “no.” Of the 53 “no” responses, 47 (42.0% of all children asked this question) accurately reported that it was the female research assistant who left, and two children inaccurately reported that it was the male research assistant.

*Did the boy leave?* Children were asked this direct question if they had not previously reported that the male had left the room. Of the 63 children who were directly asked whether or not the male research assistant left the room during the science games, 58 children (92%) accurately reported that he did not leave, and five children (8%) inaccurately reported that he left.

*Did the girl leave?* Children were asked this direct question if they had not previously reported that the female had left the room. Of the 63 who were directly asked if the female research assistant left, 24 (38%) accurately said “yes” and 39 (62%) inaccurately said “no.”

#### 3.2.2 Details About Leaving

Next, we summarize the responses from the children who accurately indicated that the female research assistant left the room and the children who inaccurately reported that either the male or female research assistant left the room.

##### 3.2.2.1 Accurate Reports of Leaving

Children's estimates of how long she left the room for (actually 3–4 min) were slightly more likely to be correct (*n* = 53; 52.5%) than incorrect (*n* = 47; 46.5%). One child did not provide a response. Most (64%) children were accurate when asked during which activity the female left (*n* = 36 correct, with an additional 29 correct, but vague in their response; *n* = 34 [34%] incorrect, *n* = 2 [1.9%] no response). When asked where they think she went, most children (*n* = 68; 67%) were accurate in reporting that she left to retrieve the geode. An additional 20 (20%) children speculated by guessing a range of activities that were typical for adults in the camp. Seven children said “I don't know,” an additional four children provided responses that were difficult to classify, and two children did not respond.

##### 3.2.2.2 Inaccurate Children

Seven children inaccurately reported that the male left the room. However, only four of these children responded to questions about the details of the male research assistant leaving. Because the male did not actually leave the room, any information the child provides will be inaccurate. We provide these details here to allow for comparison to a child reporting, by chance, the actual details of the research assistant's activities. All four children were incorrect in their estimates of how long the research assistant was gone for. Most children (*n* = 3) were inaccurate when asked during which activity the research assistant left, but one child reported the activity that took place during the female research assistant's absence, by chance. When asked where the research assistant went, two children reported a reasonable camp activity, and the other two did not respond. One 10‐year‐old child provided a particularly creative answer to the request to speculate about where the assistant went: “*To go get some free hot chocolate from the free hot chocolate room*” (unsurprisingly, there was no free hot chocolate room at camp).

## 4 General Discussion

Although children were highly accurate at reporting when an adult remained in the room for the duration of the event, many children did not report that an adult was absent when she had left the room for a substantive portion of her visit to the classroom. In the pilot study, less than a quarter of children correctly reported her absence. This rate increased in the main study, with approximately 68% of children reporting her absence; however, despite the strong salience of the female research assistant's return with the 100 lb geode, over a third of children continued to deny that she had left the room at all, even in response to the most direct question.

### 4.1 Noticing the Absence

Recall that, in the introduction, we discussed three possible reasons why a child may not report the absence of an adult: encoding failure, retrieval failure, or social pressures. As discussed earlier, in our view, the former two reasons are the most likely in the present context.

#### 4.1.1 Encoding

The difference in the rates of reporting the female's absence in the pilot compared to the main study—and how much more salient the female's departure was in the main study—support the proposition that encoding should be a central consideration. Yet, even in the main study, about a third of children still did not report her absence. Children's attention was fixated on the two visitors to the classroom, and, in the main study, the female's return with a 100 lb purple geode was an exciting event. Why, then, did so many children fail to report her absence? Encoding‐based explanations will focus on attention and retention.

Children may simply not have attended to *who* brought the geode, and instead attended to the geode itself. It is also possible that the children noticed the female bringing the geode into the room, but encoded the person delivering the geode as so unremarkable or unimportant to the upcoming activity that it was not retained in memory. Children naturally tend to be egocentric [29] and thus, are more likely to attend to details that are relevant to their current need state. While the geode might have been relevant to their interests, the deliverer of the geode may not have been. To further isolate encoding issues in future research, one could attempt to isolate encoding issues by actively instructing the children to attend to the female's activities. This is clearly an area that requires additional research.

#### 4.1.2 Retrieval

Even if the children encoded the female research assistant's absence, they may not have retrieved the information from memory when asked about it. To more effectively retrieve memories, interviewers can pose questions that better match the memory to be retrieved [30]. Perhaps a very open‐ended question could have accessed a narrative report of the female research assistant's activities that included her departure from the room. However, there is evidence that yes/no questions are more likely than open‐ended questions to elicit disclosures of minor transgressions in children [31, 32, 33], so it is unclear if an open‐ended question would have been effective in this case. We may also have used terminology that did not have our intended meaning. For example, it may be the case that asking about “leaving” and “staying” does not convey to some children what adults believe it conveys. Maybe some children consider “leaving” to be an action with permanence that does not allow for a return, or maybe an adult is considered to have “stayed” if they left and returned. We have not yet examined children's terminology related to alibi corroboration and how such understanding could impact recall. Further, perhaps a preamble is needed to justify the importance of the forthcoming corroboration questions, which could result in a more effortful memory search. We see the investigation of terminology use and framing of alibi questions as one of the most fruitful avenues for future research on children's alibi corroboration.

In the present studies, we asked questions about the research assistants’ activities in several different ways. Some questions were very direct about a particular adult's activities, whereas others were less directive. Given what we know about question types from the investigative interviewing literature [34, 35, 36], we can surmise that some of the questions posed pulled for a “yes” response (e.g., did she leave?; did she stay?) and as a result, it is more likely that children will reply with “yes.” In the future, it would be useful to experimentally explore various question phrasings to examine which would most effectively produce accurate responses. Importantly, though there were relatively few false alarms of leaving overall, *all* of the incorrect reports of leaving (both male and female) in both studies were in response to the most specific question (i.e., did she leave?; did he leave?). Thus, these findings concur with a large literature about caution when using yes/no questions [37, 38, 39].

Despite the concerns about false alarms in response to the most direct yes/no questions, it is also worth noting that many of the children's correct reports of the female leaving came in response to these same questions. In the pilot study, the large majority of correct leave responses came in response to this final, most specific question, and in the main study, almost a quarter did. This high rate is notable because children were given the opportunity to provide this information in two prior questions and were only asked each of the increasingly direct questions if they had not yet indicated that the female left. The first question (“did anyone leave”) resulted in 20% of those asked reporting the female's absence, the second question (“did both stay”) resulted in 42% of those asked reporting, and the final, most direct question (“did she leave”) resulted in 38% of those asked reporting. Thus, accurate rates of reporting the female's absence were higher in response to more direct questions. Ultimately, as with all circumstances in which adults are seeking information from children in a forensic context, interviewers must consider the balance between eliciting information using open‐ended questions that are more likely to produce accurate responses [40] and the need to obtain highly specific information that might not be produced without specific questions [41].

### 4.2 Corroborative Details

In addition to reporting that she left, the children also provided details about the female research assistant's absence. Many, perhaps most, circumstances in which an alibi is needed will only become notable after the time has passed. Thus, when encoded, the time and place of the actions of the person seeking the alibi corroboration were not experienced as noteworthy [22]. This lack of importance at the time can lead to less attention and more impoverished encoding, thus making the information more difficult to recall later.

Only about half of the children who reported the female's absence provided accurate information in response to questions about duration of absence or presumed activities. Many more details would be requested from an actual alibi corroborator (e.g., day of week, time of day, specific location information), but these initial data are consistent with data from the broader temporal detail literature and indicate that providing such information could be challenging for children [23, 25, 27].

### 4.3 Limitations and Future Directions

The present findings concur with the broader alibi literature that providing information about a person's actions—even your own prior actions—can be a difficult task [18]. However, there were also promising indications of accuracy, particularly in reports that the adult did not leave the room. This latter issue is something that has not, to our knowledge, been specifically addressed by researchers: is the ability to notice that someone is present different from the ability to notice that someone who *was* present is now absent? An important extension of this work, for both the former and the latter question, relates to the perception of the resulting evidence. Evidence is always judged by its believability, which will often ultimately be more important than its actual accuracy. Thus, an important future step is to explore how such alibis are perceived by potential triers of fact.

We did not observe age differences in children's ability to accurately notice and recall an adult's activities, but because this was not a central question in the present research, we were also not statistically powered to observe such differences. Though we do not have specific hypotheses about how well children of different ages would be able to corroborate an alibi, the developmental literature on recall of temporal information indicates that we should expect age differences to be particularly prominent up to the age of 8 years [23, 24, 25]. Future studies may seek to examine potential developmental differences and identify when children can reliably corroborate an adult's alibi.

We opted to isolate children's ability to recognize and recall the female's absence from their relationship with the accused, but this is a critical question for future research. Children will most often be alone with a person with whom they are already familiar, and thus exploring their awareness of a familiar person's activities is critical. A child may feel social pressure to lie for a familiar adult, or an adult may attempt to convince the child they were mistaken [42, 43, 44]. Further, it is likely that children who may corroborate an alibi for a familiar adult will do so within the context of a daily routine (e.g., with a parent or other caregiver). Research has clearly demonstrated that recalling individual instances of a routine can be very challenging [45]. Thus, exploring the impact of a routine or daily script on children's ability to recall an adult's activities on a particular day is essential. Relatedly, in the present design, the event was witnessed by multiple children, and, as we note in the introduction, a child's alibi witness statement will most often be required when a child is alone with an adult. Although the children in the present study were interviewed individually, they did not witness the event alone. Thus, future designs should work to create conditions that are more comparable to the situation in which a child is likely to be asked to witness an alibi.

## 5 Conclusion

Observing and reporting an adult's activities can be crucial when an adult is accused of a crime, and the child is the only potential alibi corroborator. In the present studies, we found that while most children accurately reported the presence of an adult who remained in the room for the duration of the event, many children did not report an adult's absence, and many did not accurately report details of an absence they reported. These findings raise concerns about children's ability to corroborate an alibi, but also make clear that additional research on question phrasing, terminology, and factors that influence encoding, retrieval, and social influence on reporting is needed.

## Author Contributions

H.L.P. conceptualized the study, designed the methodology, collected the data, conducted data analysis, and wrote the initial draft. A.K. contributed to conceptualization, methodological refinement, and manuscript editing. A.D.E. contributed to data analysis and manuscript editing. E.A.N. contributed to data collection, results presentation, and manuscript editing. J.W. contributed to methodological refinement and data collection.

## Conflicts of Interest

The authors declare no conflicts of interest.

## Supporting information




**Supplementary materials**: nyas70179‐sup‐0001‐Appendix.docx
