# Supplementary material for: Children as Alibi Corroborators for Adults
Source: Ann N Y Acad Sci. 2026 Jan 2;1556(1):e70179. doi: 10.1111/nyas.70179 (PMC12924167; doi:10.1111/nyas.70179)
Supplement: Supplementary file 1 — Supplementary materials: nyas70179‐sup‐0001‐Appendix.docx [file NYAS-1556-0-s001.docx]

**Appendix**

**ALIBI QUESTIONNAIRE**

PART I.

First, tell me which adults were in the room during the climate games.

PART II.

*For Part II, ask questions in order and proceed to Part III as soon as/IF the child indicates that one of the Climate Game Leaders left the room during the climate games*.

(i) Did anyone leave the room during the climate games? YES/NO

If “yes,” who left the room?

(ii) There were two people who taught you the climate games. Did both of the people stay in the room the whole time you did the climate games? YES/NO

If “no,” who left the room?

(iii) Did the boy who taught you the climate games leave the room? YES/NO

(iv) Did the girl who taught you the climate games leave the room? YES/NO

PART III.

*Only ask Part III questions if the child indicates that one or more of the Climate Game leaders left the room*.

(v) How long was s/he gone for?

*If the child says “I don’t know” ask:*

Can you estimate how long s/he was gone for?

(vi) What was happening in the room when s/he left?

(vii) Where do you think s/he went?
